# Bio-detheobromination of cocoa pod husks: reduction of ochratoxin A content without change in nutrient profile

**DOI:** 10.1186/s12934-018-0931-x

**Published:** 2018-05-19

**Authors:** Daniel Oduro-Mensah, Augustine Ocloo, Sammy T. Lowor, Cheetham Mingle, Laud K. N.-A. Okine, Naa Ayikailey Adamafio

**Affiliations:** 1 Department of Biochemistry, Cell and Molecular Biology, School of Biological Sciences, University of Ghana, Accra, Ghana; 2 Physiology/Biochemistry Division, Cocoa Research Institute of Ghana, New Tafo-Akim, Ghana; 3 Food Physicochemical Laboratory, Food and Drugs Authority, Accra, Ghana; 4 Department of Applied Chemistry and Biochemistry, Faculty of Applied Sciences, University for Development Studies, Navrongo Campus, Navrongo, Ghana

**Keywords:** *Aspergillus*, Cocoa pod husk, Ochratoxin A, *Talaromyces*, Theobromine

## Abstract

**Background:**

Utilization of cocoa pod husks (CPH) in animal feed is hindered by the presence of theobromine, which is variably toxic to animals. Treatment of this agro-waste to remove theobromine, while preserving its nutrient content, would allow beneficial use of the millions of metric tonnes discarded annually. The aim of this study was to assess the suitability of selected theobromine-degrading filamentous fungi for use as bio-tools in degradation of theobromine in CPH.

**Results:**

The candidate fungi assessed in this study were an *Aspergillus niger* (AnTD) and three *Talaromyces* spp. (TmTD-1, TmTD-2, TvTD) isolates. All the fungi eliminated CPH theobromine, 0.15% w/w starting concentration, within 7 days of start of treatment, and were capable of degrading caffeine and theophylline. The fungi decreased CPH ochratoxin A content by 31–74%. Pectin was not detectable in fungus-treated CPH whereas parameters assessed for proximate composition were not affected.

**Conclusions:**

The data provide ample evidence that the four isolates can be applied to CPH for the purpose of eliminating theobromine and decreasing ochratoxin A content without affecting nutrient profile. Comparatively, *Talaromyces verruculosus* TvTD was considered as most suitable for use as a bio-tool in detheobromination of CPH for animal feed.

## Background

In cocoa and coffee-producing countries, the major agro wastes generated include cocoa pod husks, cocoa bean shells and coffee bean shells. There is huge potential for use of the wastes in production of cheap animal feed following appropriate treatment to remove or reduce their methylxanthine content. For cocoa-growing countries, the major by-product of the cocoa industry is the cocoa pod husk (CPH). By weight, there is an estimated 7–8 kg of fresh CPH for every 1 kg of cocoa beans produced [1, 2]. With the enormous quantities discarded annually, CPH remains an untapped resource that could be of significant agricultural and economic benefit.

Incorporation of CPH in animal feed is a promising application of keen interest [3–5]. Over the years, however, studies have shown that acceptability of CPH by different animals ranges between 1.9 and 25% of animal feed ([6–8]. Tolerance of CPH in feed is limited by problems with digestibility, palatability and adverse effects of some of its chemical constituents [8, 9]. Whereas the challenges with CPH digestibility have been linked to its anti-nutrient content, the problems with palatability and adverse chemical effects have mainly been linked to the theobromine content [8, 10]. Theobromine is the principal methylxanthine accumulated by the cocoa plant and in CPH, theobromine content is approximately 0.15–0.4% w/w [8, 10].

The European Union limit on theobromine content of animal feed is 300 mg/kg [8], which translates into approximately 13.7% w/w CPH in animal feed. Theobromine is reported to exert deleterious effects on animal physiology, including retarded growth and lethargy in pigs, reduced milk yield in cattle and delayed egg-laying in chicken [8, 11–14]. Caffeine and theophylline, whose effects are similar but not limited to effects of theobromine in animals [15, 16], have also been reported at relatively lower levels in CPH [17, 18]. Existing approaches for removal of theobromine from cocoa material have accompanying high costs, *vis a vis* chemicals and/or reagents used, solvent volumes and energy requirements, and usually result in decreased nutritional value of treated material [19, 20]. Whereas detheobromination of up to 72% has been reported for methods based on the existing approaches, accompanying losses of soluble nutrients, including up to 12.6% protein loss, have been documented [20].

Other components of CPH include pectin and anti-nutrients such as lignin and tannin. In animals, dietary pectin is reported to be advantageous: aiding digestibility, stimulation of protein metabolism and utilisation of metabolisable energy [21, 22]. In contrast, the major limiting factor of fiber digestibility in farm animals, including ruminants, is the lignin content. Cocoa pod husks contain approximately 14% lignin by dry weight [9]. Lignin binds strongly to hemicellulose and cellulose, making them unavailable as nutrients [23]. Similarly, dietary tannins limit protein bioavailability and digestibility. High molecular weight polyphenols, which are generally designated as tannins, have strong affinities for proteins, forming insoluble protein-tannin complexes [24, 25]. Also of interest is ochratoxin A which is frequently found to contaminate cocoa-associated material [26, 27]. Like theobromine, ochratoxins are toxic to animals, exhibiting immunosuppressive, nephrotoxic, teratogenic, and carcinogenic properties [28, 29].

In view of the multiple factors limiting CPH utilization as animal feedstuff, therefore, remediation approaches that may be capable of resolving more than challenge, with minimal adverse impact on CPH nutritional value, would be of huge advantage. Detheobromination using a filamentous fungus as bio-tool has been shown to be a plausible alternative [30]. In a previous study, isolation and characterization of several filamentous fungal isolates capable of theobromine degradation were reported [31]. As a furtherance, the current study was aimed at assessing and comparing the suitability of four of the theobromine-degrading fungi for biodetheobromination of CPH. The four fungi were selected on the basis of being the most efficient at theobromine degradation, and their potential for use in detheobromination of CPH without any requirements for modification of ambient temperature or pH of the substrate [31]. In the current study, evaluation of suitability of the isolates for detheobromination of CPH was based on (1) effect on CPH theobromine content, (2) effect on CPH ochratoxin A content, (3) effect on nutrient content of CPH and (4) documented pathogenicity potential of isolate(s).

## Methods

### Preparation of CPH substrate

Fresh cocoa (*Theobroma cacao*) pod husks were collected immediately after pod-breaking of ripe fruits at the Cocoa Research Institute of Ghana (CRIG), Akim-Tafo, Ghana. Husks to be used as substrate for fungal cultures were sun-dried for up to 2 weeks, milled to approximately 2 mm particle size and autoclaved (121 °C and 15 psi for 20 min).

### Study isolates

Four filamentous fungi previously confirmed as capable of theobromine-degradation [31] were used as study isolates. The isolates were *Aspergillus niger* AnTD, *Talaromyces verruculosus* TvTD and two *Talaromyces marneffei* TmTD-1 and TmTD-2, with DNA identification sequences curated as KY697093, KY697096, KY697104 and KY697103, respectively, in the GenBank database [31].

### Preparation of fungal inoculum

A spore suspension from each isolate was prepared in distilled water [32] after incubation for up to 14 days on theobromine-sucrose agar slants at room temperature (25–29 °C). For use as inoculum, the spore suspensions were standardised to 2 × 10^6^ spores/ml.

### Colonization of CPH

To assess ability of each isolate to colonize CPH, approximately 10 g of milled, sterilized CPH in 35 mm petri dishes were inoculated with spore suspension (10 µl per gram), and moistened with distilled water at a ratio of 1:1 (w/v). The setups were incubated at room temperature for 7 days, and were visually observed for fungal growth at the end of the period.

### Biodetheobromination: effect of moisture content

Moisture content of CPH that would be most suitable for in situ theobromine degradation by the isolates was investigated at 1:1, 1:2 and 1:3 w/v moisture levels. For each isolate, approximately 50 g of CPH was inoculated with spore suspension as described. Control samples were inoculated with inoculum inactivated by autoclaving. Treatment flasks were incubated at room temperature for 7 days, autoclaved and dried to constant weight at 50 °C.

### Extraction of CPH theobromine

Theobromine content of CPH was extracted by a modification of the method for extraction of theobromine from cocoa beans (Method 980.14, [33]). Briefly, approximately 5 g of milled CPH was defatted with two portions of 30 ml petroleum ether (BDH; Poole, England) at room temperature, and left overnight in a fume hood to dry. The dried material was boiled in 100 ml of distilled water for 20 min. After the period, an extra 50 ml distilled water was added and the material was boiled for a further 20 min. Another 50 ml of distilled water was added and the material was boiled again for 20 min. Between 70 and 90 ml of supernatant from each sample was concentrated by rotary evaporation under reduced pressure at 50 °C, filtered through a Whatman Spartan syringe filter with a 0.45 µm membrane (Sigma Aldrich; St. Louis, MO) and analyzed for theobromine by HPLC.

### HPLC quantification of CPH theobromine content

Chromatographic separation was done at 27 °C on an Atlantis dC18 5 µm column (part no. 186001344) fitted to a Waters HPLC unit consisting of a Waters In-Line degasser AF, Waters 1525 binary HPLC pump and Waters 2487 dual wavelength absorbance detector set to monitor at 270 nm. Gradient elution at 1.5 ml/min was by a solvent system of acetonitrile (Sigma-Aldrich; St. Louis, MO) and 0.1% formic acid (BDH, England). The formic acid solution was adjusted to pH 3.75 with ammonia (BDH; Poole, England). Mobile phases were pumped according to the gradient profile in Table 1. Solutions of theobromine, as well as theophylline and caffeine (Sigma-Aldrich; St. Louis, MO) were prepared and used as standards.

**Table 1:** Gradient elution profile for separation of theobromine and its metabolites

Time (min)	Eluent mix (%)		Flow rate (ml/min)
	0.1% ammonium formate	Acetonitrile	
0	98	2	1.5
6	95	5	1.5
9	98	2	1.5
10	98	2	1.5
11	92	8	1.5
14	88	12	1.5
17	89	11	1.5
18	98	2	1.5

### Ochratoxin A content of CPH

Effect of fungal treatment of CPH on OTA content was investigated by inoculating milled CPH at a moisture ratio of 1:3 w/v for each isolate. CPH samples moistened and inoculated with inactive inoculum were used as controls. Cultures were incubated for 7, 10 or 20 days, respectively. At the end of incubation, each sample was autoclaved and dried to constant weight. Concentration of OTA was determined using reagents from a MaxSignal OTA test kit (Bioo Scientific; Austin, TX). Absorbances were read at 450 nm on a microtiter plate reader.

### CPH pectin and polyphenol degradation by isolates

Pectin was extracted from approximately 15 g of dried, control or fungus-treated CPH samples that had been steeped in 85% ethanol at 70 °C for 20 min [34]. Pectin content of each sample was determined gravimetrically from precipitates obtained after the extraction procedure.

Total polyphenol content of each CPH sample was extracted [35] and measured [36]. Concentration of tannin in each sample was determined after treating a portion of polyphenol extract with polyvinyl polypyrrolidone (Sigma Aldrich; St. Louis, MO.). Solutions of gallic acid (5–20 µg/ml) were used as standards and polyphenol concentrations were expressed as gallic acid equivalents.

### Proximate analysis of control and fungus-treated CPH

Samples of CPH, either control or fungus-treated for up to 10 days, at moisture ratio of 1:3 w/v, were autoclaved, dried to constant weight and analyzed for proximate composition. Dry matter was determined by method 930.15 [37]. Crude protein and total fat were determined by methods 942.05 and 920.39, respectively [37]. Ash was determined according to method 695.17 [37], after combustion in a muffle furnace at 600 °C. All fibers were determined inclusive of residual ash. Acid detergent fiber (ADF), neutral detergent fiber (NDF) and acid detergent lignin (ADL) were determined [38]. Cellulose was calculated as the difference between ADF and ADL [39].

### Degradation of caffeine and theophylline by isolates

Each isolate was cultured in 20 ml theobromine liquid medium, containing 1.9 mM theobromine at pH 5.8 [31] and inoculated to contain 2 × 10^4^ spores per ml of medium. Control cultures were inoculated with inactivated inoculum. Cultures were incubated with agitation at room temperature. After 3 days, caffeine and theophylline were added as substrates to each culture at final concentrations of approximately 0.08 and 0.1 mM, respectively, and incubated for a further period of 1.5 h. Aliquots from each culture were filtered and analyzed for caffeine and theophylline by HPLC as described.

All statistical analyses were performed with data analyses tools in Microsoft Excel version 14, Microsoft Office Professional Plus 2010. For determining significance of differences, data were analyzed by one-way ANOVA and post hoc by two-tailed t test. Significance analyses were conducted at α = 0.05.

## Results

**Fig. 1:**
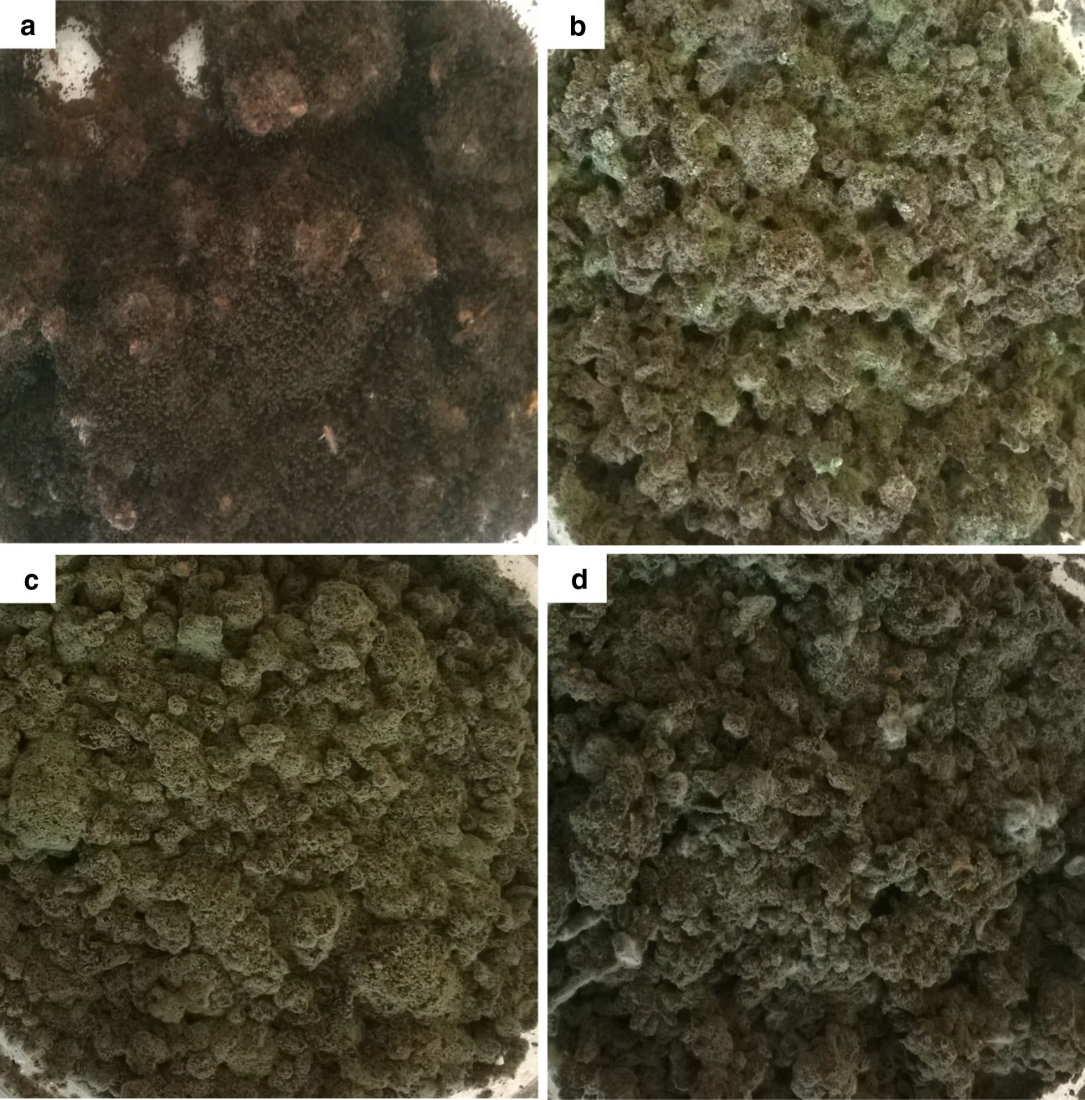
Cocoa pod husks colonized by **a** *Aspergillus niger* AnTD, **b** *Talaromyces marneffei* TmTD-1, **c** *Talaromyces verruculosus* TvTD and **d** *Talaromyces marneffei* TmTD-2. Milled CPH in petri dishes were inoculated with spore suspensions of selected isolates at a moisture ratio of 1:1 and incubated at room temperature for 7 days

To evaluate suitability of the four selected fungal isolates for use in treatment of CPH for animal feed, experiments were conducted to assess the ability of each isolate to colonize CPH. Visual observation showed that each isolate had readily colonized the entire exposed surface of the pod husks within 5 days (Fig. 1a–d).

**Fig. 2:**
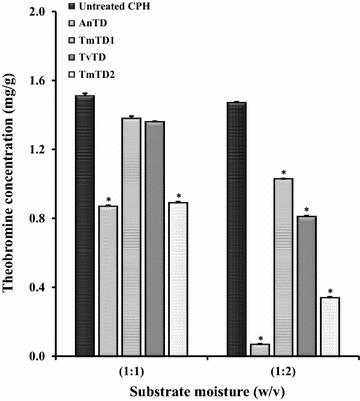
Effect of fungal treatment on theobromine content of CPH. Fungal isolates were grown for 7 days at ambient temperature on CPH at different moisture levels. Theobromine was extracted from CPH samples and quantified by HPLC. Each bar represents mean ± SEM of n = 3. *Significantly different from untreated (control) CPH (p < 0.05). *AnTD Aspergillus niger*, *TmTD1 Talaromyces marneffei* 1, *TvTD Talaromyces verruculosus*, *TmTD2 Talaromyces marneffei* 2

**Fig. 3:**
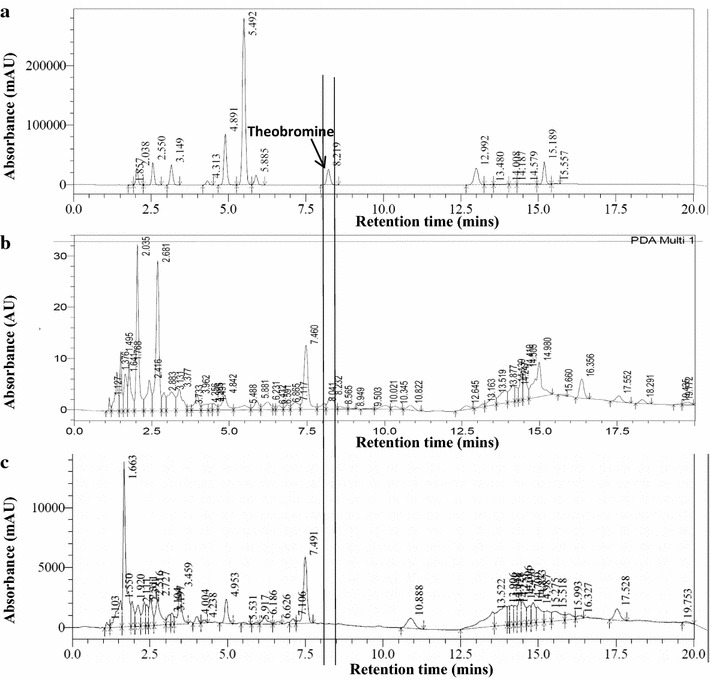
HPLC chromatograms of **a** A mixture of methylxanthines, and aqueous extracts of **b** control and **c** fungus-treated sterilized CPH. A vertical line through all the plots indicates the expected elution time for theobromine. CPH was treated with TvTD for 10 days. Aqueous extracts of CPH were scanned for all absorbing compounds in the range 190–400 nm

The use of different levels of moisture for CPH treatment revealed that moisture content had a pronounced effect on biodetheobromination by the isolates (Fig. 2). Theobromine content of CPH was approximately 1.51 mg/g. At substrate moisture contents of 1:1 and 1:2 w/v, the isolates degraded at least 8.6 and 49% of CPH theobromine, respectively. At substrate moisture 1:3 (w/v) however, theobromine was not detectable in any of the treated CPH samples. Representative HPLC chromatograms of extracts from control and fungus-treated CPH, indicating absence of the theobromine peak post-treatment, are shown in Fig. 3a–c.

**Fig. 4:**
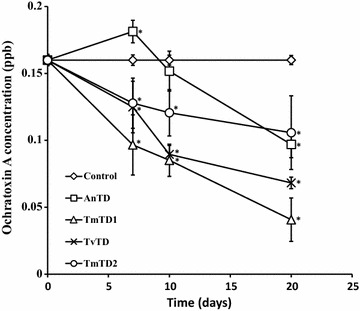
Effect of treatment of CPH on ochratoxin A concentration. The treatment cultures were incubated at room temperature. Ochratoxin A levels were determined using a MaxSignal Ochratoxin A test kit. Each point represents mean ± SEM of n = 3. *Significantly different (p < 0.05) from baseline value (0.16 ppb). *AnTD Aspergillus niger*, *TmTD1 Talaromyces marneffei* 1, *TvTD Talaromyces verruculosus*, *TmTD2 Talaromyces marneffei* 2

Baseline concentration (0.16 ppb) of OTA in CPH controls did not change with time whereas OTA levels in all fungi-treated CPH samples had declined significantly (p < 0.05) at termination of treatment (Fig. 4). However, AnTD recorded a slight increase, from 0.16 to 0.18 ppb, in OTA concentration on day 7, before declining on subsequent days.

**Fig. 5:**
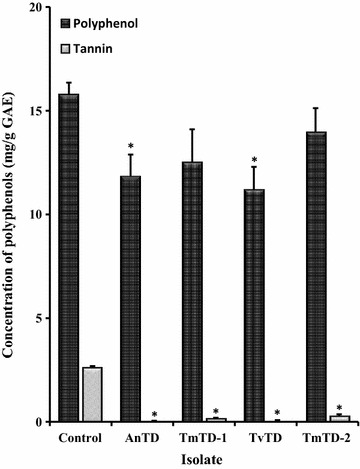
Effect of treatment on levels of total polyphenols and tannins in CPH. Milled CPHs were treated with candidate isolates for 7 days. At the end of the period, the treated materials were autoclaved, dried and analysed for total polyphenol and tannin levels in gallic acid equivalents (GAE). Each bar represents mean ± SEM of n = 3. *Significantly different (p < 0.05) from control

Control CPH had pectin content of approximately 7.65% w/w whereas in all cases, pectin was not measurable in fungus-treated CPH (data not shown). Comparison of total polyphenol and tannin levels indicated that relative to the control CPH, total polyphenol levels decreased by ≥ 11% whereas tannin concentration decreased by ≥ 89% (Fig. 5). Proximate analyses revealed no significant changes (p > 0.05) in the levels of total protein, crude fiber, total fat, acid detergent fiber, neutral detergent fiber, cellulose, hemicellulose, lignin and total ash between untreated and fungus-treated CPH at days 7 or 10 of treatment (Table 2).

**Table 2:** Proximate composition of untreated and fungus-treated CPH

Nutrient	% DM of untreated CPH	% DM of treated CPH							
		AnTD		TmTD1		TvTD		TmTD2	
		Day 7	Day 10	Day 7	Day 10	Day 7	Day 10	Day 7	Day 10
Crude protein	5.3 ± 0.1	5.4 ± 0.1	5.3 ± 0.1	5.3 ± 0.1	5.4 ± 0.1	5.4 ± 0.1	5.1 ± 0.1	5.1 ± 0.1	5.1 ± 0.1
Crude fiber	41.0 ± 0.1	40.4 ± 0.5	39.8 ± 0.3	39.0 ± 0.2	41.2 ± 0.1	41.2 ± 0.2	40.6 ± 0.7	40.6 ± 0.3	40.5 ± 0.3
Fat	0.5 ± 0.1	0.5 ± 0.1	0.5 ± 0.1	0.5 ± 0.1	0.8 ± 0.1	0.8 ± 0.1	0.8 ± 0.1	0.8 ± 0.1	0.8 ± 0.1
ADF	57.8 ± 0.8	58.6 ± 0.2	58.9 ± 0.4	58.9 ± 0.2	59.6 ± 0.7	60.6 ± 0.9	58.4 ± 0.8	58.4 ± 0.2	57.8 ± 0.3
NDF	66.6 ± 0.4	66.3 ± 0.1	66.8 ± 0.9	66.8 ± 0.3	64.9 ± 0.2	64.9 ± 0.6	65.7 ± 0.6	65.7 ± 0.6	66.8 ± 0.4
Cellulose	28.6 ± 0.3	28.3 ± 0.3	28.3 ± 0.1	28.3 ± 0.2	29.5 ± 0.4	29.5 ± 0.2	29.9 ± 0.4	29.9 ± 0.6	29.0 ± 0.3
ADL	29.4 ± 0.7	30.4 ± 0.4	31.4 ± 0.2	31.4 ± 0.1	30.6 ± 0.3	30.6 ± 0.6	30.3 ± 0.5	30.3 ± 0.5	29.4 ± 0.2
Total ash	9.7 ± 0.1	9.8 ± 0.1	9.8 ± 0.1	9.8 ± 0.1	9.7 ± 0.1	9.7 ± 0.1	9.6 ± 0.1	9.6 ± 0.1	9.7 ± 0.1

*%DM* Percentage of dry matter, *ADF* acid detergent fiber, *NDF* neutral detergent fiber, *ADL* acid detergent lignin, Values shown represent mean ± SEM of n = 3

**Fig. 6:**
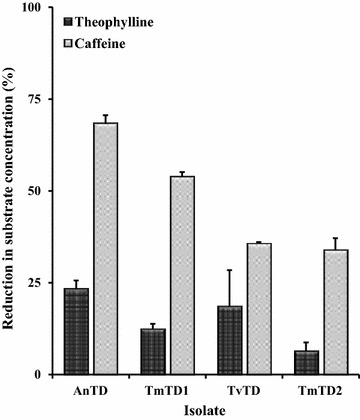
Degradation of theophylline and caffeine by theobromine-degrading fungal isolates. The isolates were cultured with agitation for 3 days. After the period, substrates were added to the whole cultures and incubation continued with agitation for 90 min. Plot shows percent reduction in baseline substrate concentration (0.1 mM theophylline; 0.08 mM caffeine) for each fungal isolate. Each bar represents mean ± SEM for n = 4. *AnTD Aspergillus niger*, *TmTD1 Talaromyces marneffei* 1, *TvTD Talaromyces verruculosus*, *TmTD2 Talaromyces marneffei* 2

Data from the experiments to investigate degradation of caffeine and theophylline indicated that the isolates degraded both compounds to varying degrees (Fig. 6). Also, there were no peaks on HPLC chromatograms corresponding to accumulation of degradation intermediates (data not shown).

## Discussion

In the first report on biodetheobromination, [30] achieved 72% theobromine removal from CPH after 7 days of treatment using *A. niger* as bio-tool. Subsequently, a number of reports have indicated successful detheobromination of CPH and cocoa meal, using isolates of *A. niger* or *A. awamori* [40–42]. Isolates of these two *Aspergillus* spp., however, are known producers of OTA [43] and so could increase OTA content of substrates. In this study, criteria used to assess suitability of the selected fungi for treatment of CPH, potentially for use as animal feed, were: (1) ability to colonize CPH, (2) detheobromination efficacy, (3) effect on CPH ochratoxin A content, (4) effect on proximate composition of CPH and (5) pathogenicity potential.

Ability of the selected fungi to colonize milled CPH was successfully demonstrated under solid-state conditions at ambient temperature. This is important for application of the fungi because the spread of hyphae throughout a substrate is key to overall efficacy of bioconversion/bioremediation of substrate. The milling of CPH was to increase surface area for fungus-substrate interaction, and to allow for formulation of fungus-treated CPH into feed material suitable for testing on laboratory rodents. With reference to the different substrate moisture contents tested, only the highest (1:3 w/v) resulted in complete degradation of CPH theobromine by all of the isolates within 7 days. It would, however, be advantageous to keep moisture levels low in order to minimise the cost of post-treatment drying of treated material. Hence, moisture levels beyond 1:3 w/v and/or shorter treatment periods were not tested. The absence of differences between HPLC profiles of sterilized and unsterilized TvTD-treated CPH suggested that there was no effect on the treated CPHs due to exposure to heat during the sterilization process.

Generally, fungal treatment resulted in decreased OTA content of CPH, from approximately 0.16 ppb (control) to 0.03–0.09 ppb at termination (day 20). Only the *Talaromyces* spp. isolates had significantly decreased OTA content of CPH by day 7 of treatment (Fig. 4). The slight increase, from 0.16 to 0.18 ppb, recorded for AnTD on day 7 suggests that the *A. niger* isolate used in the study may have been ochratoxigenic. There was no indication, however, that the *Talaromyces* spp. isolates were ochratoxigenic. Moreover, OTA production is currently known to be limited to a few species of the *Aspergillus* and *Penicillium* genera [44]. Woody materials such as CPH have characteristic high C/N ratios [45]. This makes such material growth-limiting, because nitrogen availability is low. Hence, it may be that microbes growing in such environments attempt to utilize any nitrogen sources present. This may explain why OTA, which is a nitrogen-containing compound, was found to decrease with duration of treatment. This suggests also that the *Talaromyces* spp. isolates synthesise enzymes capable of degrading OTA. Significantly, the findings indicate that with reference to OTA content, ingestion of fungus-treated CPHs might be less detrimental to the health of animals than control CPH. It must be noted however that the OTA levels detected in the CPH used were approximately three orders of magnitude below the highest permissible limits [46].

A comparison of total polyphenol and tannin levels in control and fungus-treated CPH indicated that the isolates could degrade some CPH polyphenols. Digestibility of feed ingredients is reported to be inversely related to polyphenol content [47, 48]. However, the marginal reduction in total polyphenol level makes it unlikely that treatment with the isolates, as described, would result in any tangible increase in CPH digestibility. Nevertheless, the near elimination of tannin could be advantageous to nutrient value of fungus-treated CPH *vis a vis* availability and utilization of dietary protein. In contrast, the complete elimination of pectin by the fungi might have implications for the formulation of CPH based feed for non-ruminants since pectin is reported to aid in digestibility and nutrient utilization [21, 22].

Proximate analysis of fungus-treated CPH did not reveal any significant differences in crude protein, crude fibre, crude fat, ADF, ADL, cellulose and total ash levels relative to control CPH (Table 2). Although treatment of CPH as described eliminated theobromine from the substrate, nutrient profile was generally unaffected, contrary to expectations. Regarding protein content, for example, an increase in value post-treatment was anticipated. This is because the fungi were expected to secrete at least some of the enzymes needed for degradation of theobromine [31] and any other macromolecules they may have utilised. Therefore, total nitrogen content in the treated cocoa husk was expected to be significantly higher than the control. The data suggested, however, that (1) CPH protein utilized for growth by the fungi was equivalent to that replaced by secretion or (2) not enough protein may have been secreted by the fungi to increase protein content of treated CPH or (3) reflecting ability of the fungi to grow and degrade theobromine in nutrient-poor media [31], insignificant amounts of protein may have been utilized for growth on CPH. Incubation was extended to 10 days as a precautionary measure to ensure that theobromine would have been completely removed from CPH at the end of treatment. Longer treatments periods, which may have resulted in modification of nutrient content were, however, not investigated due to the possibility of production of undesirable metabolites by the isolates.

To further aid in selection of one isolate for treatment of CPH, experiments were designed to assess the isolates with respect to ability to degrade other methylxanthines (caffeine and theophylline) present in cocoa material [17, 18]. Substantial reductions in levels of caffeine and theophylline occurred when added to cultures of the theobromine-degrading fungi in liquid medium. Although there was no direct evidence of demethylation or oxidation of either substrate, there were no peaks corresponding to accumulation of downstream metabolites on chromatograms. This indicates that enzyme systems of the isolates are capable of completely degrading both compounds, similar to theobromine. The results further suggest that all the four fungi may secrete broad specificity methylxanthine-degrading enzyme systems, or may have the capacity to produce specific demethylases/oxidases for each methylxanthine. Clearly, these fungi are versatile methylxanthine degraders which might prove useful not only for the elimination of theobromine, caffeine and theophylline from cocoa waste products but also for the bioremediation of other agro-residues such as coffee husks, kola husks and shea nuts/husks which contain theobromine as well as other methylxanthines [49].

In most aspects of their interactions with theobromine [31] and CPH, the fungi compared favorably with each other. However, the *A. niger* isolate, AnTD, demonstrated potential for OTA-production on CPH, besides being potentially pathogenic [50]. Also, the *T. marneffei* isolates, TmTD-1 and -2, were considered as potentially pathogenic, although due to their demonstrated detheobromination potential [31], it was desirable to characterise them *vis a vis* biodetheobromination of CPH. *Talaromyces marneffei* is a species rapidly emerging as a human pathogen, especially for immunocompromised individuals [51, 52]. Field application of the *T. marneffei* isolates, as biodetheobromination agents, would necessitate stringent containment measures that could increase production costs considerably. In contrast, *T. verruculosus* is not known to be a human pathogen. TvTD was, therefore, selected as the choice isolate for use as a bio-tool in detheobromination of CPH.

## Conclusions

This study reports the elimination of theobromine from CPH by fungal treatment. Overall, the data show that the isolates can be applied at ambient temperature to colonize CPH and degrade theobromine, as well as caffeine and theophylline, in situ. With reference to OTA content, fungus-treated CPH may be safer than untreated CPH for animal consumption. In view of the ochratoxigenicity and/or potential pathogenicities of the study isolates, *T. verruculosus* TVTD is recommended as a bio-tool for detheobromination as well as removal of other methylxanthines from substrates.

## Abbreviations

AnTD: *Aspergillus niger* AnTD KY697093
CPH: cocoa pod husk
GAE: gallic acid equivalent
HPLC: high performance liquid chromatography
OTA: ochratoxin A
ppb: parts per billion
TmTD-1: *Talaromyces marneffei* KY697096
TmTD-2: *Talaromyces marneffei* KY697104
TvTD: *Talaromyces verruculosus* KY697103
